# Outcomes of Silastic Septal Button Insertion for Nasal Septal Perforations: A Five-Year Retrospective Clinical Audit

**DOI:** 10.7759/cureus.92732

**Published:** 2025-09-19

**Authors:** Alicia Wong, Mostafa Mehana, Hisham Khalil

**Affiliations:** 1 Otolaryngology, University Hospitals Plymouth NHS Trust, Plymouth, GBR

**Keywords:** nasal septal perforation, otolaryngology, rhinology, silastic septal button, symptom control

## Abstract

Introduction

Nasal septal perforations can cause morbidity through crusting, whistling, epistaxis, and nasal obstruction. Management options include conservative therapy, surgical closure, or septal button insertion. At our institution, surgical closure is generally not offered for perforations greater than 2 cm in diameter or when the nasal mucosa remains unhealthy despite optimal medical therapy. This audit evaluated outcomes of silastic septal button insertion at a tertiary care centre over a five-year period.

Methods

A retrospective clinical audit was conducted among patients who underwent prefabricated silastic septal button insertion between 2019 and 2024 at University Hospitals Plymouth NHS Trust, United Kingdom. Demographic data, presenting symptoms, and management outcomes were extracted from clinical records. A PubMed literature review was performed to contextualise findings.

Results

Seventy-seven patients (37 females, 40 males; mean age 46.8 years, median 48 years) underwent silastic septal button insertion. Presenting symptoms included nasal crusting, whistling, epistaxis, obstruction, nasal discharge, facial pain, headache, postnasal drip, and altered olfaction. No further intervention was required in 39/77 patients (50.6%). A single replacement was required in 20/77 (26.0%), multiple replacements in 4/77 (5.2%), button removal in 12/77 (15.6%), and adjustment without replacement in 2/77 (2.6%). Complications included local infection (7.8%), granulation tissue (5.2%), and button migration (2.6%). All patients reported a positive surgical experience; 63/77 (81.8%) described improved quality of life, and 72/77 (93.5%) said they would recommend the procedure.

Conclusion

Silastic septal button insertion appears to be a safe and practical management option for patients with symptomatic nasal septal perforations who are unsuitable for or decline surgical closure. While many patients experienced satisfactory symptom control without further intervention, a substantial proportion required replacement, adjustment, or removal. Given the retrospective, single-centre nature of this audit and its inherent limitations, these results should be interpreted with caution. Careful counselling remains essential to set expectations regarding the potential need for ongoing management. Larger, prospective multicentre studies with standardised outcome measures would help clarify the long-term role of septal buttons in clinical practice.

## Introduction

A nasal septal perforation is a full-thickness defect of the cartilaginous or bony septum, resulting in an abnormal communication between the two nasal cavities. Causes include trauma, prior nasal surgery, chronic inflammatory conditions such as granulomatosis with polyangiitis, infections, iatrogenic injury (e.g., excessive cautery), malignancy, and cocaine use [1,2]. Perforations may alter nasal airflow dynamics [3] and present variably: some patients remain asymptomatic, while others develop troublesome symptoms such as nasal discharge, crusting, whistling, obstruction, postnasal drip, facial pain, headache, epistaxis, or altered olfaction [2,4].

Management depends on perforation size, location, mucosal health, underlying aetiology, and patient preference. Conservative measures, such as humidification or emollients, may alleviate mild symptoms but do not close the defect. Surgical closure offers definitive treatment but may be unsuitable for large perforations (>2 cm) or in patients with poor mucosal quality [5].

Septal buttons, first introduced in the 1970s, emerged as a practical alternative. Medical-grade silicone proved especially useful due to its comfort, biocompatibility, ease of insertion, and ability to occlude the defect, thereby reducing symptoms from airflow turbulence [6]. Modern designs have further improved flexibility, softness, and size variability, enhancing patient tolerance.

Despite their widespread use, evidence on the long-term efficacy, complication rates, and patient-reported outcomes of septal buttons remains limited. They are often considered a second-line option when surgical closure is not feasible. This audit aims to evaluate outcomes and patient experience with silastic septal button insertion at a United Kingdom tertiary care institution over a five-year period.

## Materials and methods

This retrospective clinical audit included all patients who underwent prefabricated silastic septal button insertion at University Hospitals Plymouth NHS Trust between January 2019 and October 2024. Eligible patients had symptomatic nasal septal perforation, had trialled and failed medical management, and attended at least one documented postoperative follow-up. Of 78 identified cases, one was excluded due to irretrievable records, leaving 77 for analysis.

Electronic health records were reviewed to extract demographic data, presenting symptoms, and outcomes. Perforation size was not consistently documented and was therefore excluded from analysis; all perforations were anterior. Procedures were performed under general anaesthesia, with prefabricated silastic buttons trimmed intraoperatively to match the perforation. During follow-up, buttons were adjusted, modified, or removed as needed to ensure fit. Patients were routinely reviewed at four weeks, with further follow-up arranged if clinically indicated.

Patient-reported outcomes were captured retrospectively from clinic letters and records, including satisfaction, perceived quality-of-life improvement, and whether patients would recommend the procedure. No validated symptom or QoL instruments (e.g. SNOT-22, NOSE scale) were used. Where follow-up data were incomplete, patients were contacted by telephone to obtain missing information.

Data were analysed descriptively. Continuous variables were summarised as means with standard deviations or medians with interquartile ranges, as appropriate; categorical variables were expressed as frequencies and percentages. Confidence intervals for proportions were calculated using the Wilson score method in Microsoft Excel (Microsoft Corp., Redmond, WA, USA). Subgroup analysis, regression modelling, and time-to-event analysis were not performed, as consistent patient-level data on perforation size, comorbidities, and follow-up duration were unavailable.

To contextualise findings, a PubMed search was performed on October 31, 2024, using the terms (“nasal septal perforation” OR “septal perforation”) AND (“silastic button” OR “septal button”). The search was restricted to English-language studies in adult populations, with a 10-year date limit. Reference lists of relevant studies were also screened.

Ethics statement

This study was conducted and approved as a retrospective audit at University Hospitals Plymouth NHS Trust, in accordance with local governance requirements. As silastic septal button insertion was routine clinical care, formal research ethics committee approval and individual patient consent were not required.

## Results

A total of 77 patients met the inclusion criteria, comprising 37 females and 40 males, with a mean age of 46.8 years (median 48 years). Presenting symptoms included nasal crusting, whistling, epistaxis, obstruction, nasal discharge, facial pain, headache, postnasal drip, and altered olfaction. Perforation diameter was not consistently recorded; however, all perforations were located anteriorly (77/77, 100%). 

**Figure 1:**
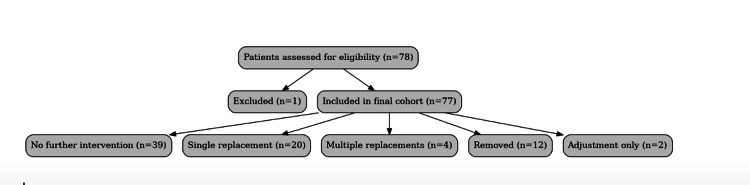
Flow diagram showing patient inclusion and outcomes. Seventy-eight patients were initially identified; one was excluded due to irretrievable clinical records, leaving 77 patients for the analysis. Subsequent outcomes following silastic septal button insertion are detailed, including rates of no further intervention, single or multiple replacements, removal, and adjustment only.

Procedural outcomes are summarised in Figure 1, with detailed frequencies shown in Table 1. Following insertion, no further intervention was required in 39/77 patients (50.6%, 95% CI 39.7-61.5%). A single replacement was needed in 20/77 (26.0%, 95% CI 17.5-36.7%), while multiple replacements were required in 4/77 (5.2%, 95% CI 2.0-12.6%). Button removal occurred in 12/77 patients (15.6%, 95% CI 9.1-25.3%), most commonly due to discomfort (5/12), infection (4/12), or extrusion (3/12). Adjustment without replacement was necessary in 2/77 patients (2.6%, 95% CI 0.7-9.0%).

**Table 1:** Outcomes of silastic septal button insertion in 77 patients with nasal septal perforation. Frequencies and percentages are presented with corresponding 95% confidence intervals (CIs). Percentages are calculated using the total cohort (n = 77) as the denominator.

Outcome	Patient frequency (n)	% (95% CI)
No further intervention	39	50.6 (39.7-61.5)
Single replacement	20	26.0 (17.5-36.7)
Multiple replacements	4	5.2 (2.0-12.6)
Button removed	12	15.6 (9.1-25.3)
Adjustment only	2	2.6 (0.7-9.0)

Documented complications included localised infection in 6/77 patients (7.8%, 95% CI 3.6-16.0%), granulation tissue in 4/77 (5.2%, 95% CI 2.0-12.6%), and button migration in 2/77 (2.6%, 95% CI 0.7-9.0%). No cases of septal enlargement were observed.

Patient-reported outcomes were favourable. All patients (77/77, 100%) described a positive surgical experience. Documented improvement in quality of life was noted in 63/77 patients (81.8%), and 72/77 (93.5%) indicated they would recommend septal button insertion to others with similar symptoms.

## Discussion

This single-centre retrospective audit indicates that silastic septal button insertion is a feasible and generally well-tolerated option for managing symptomatic nasal septal perforations. In our cohort, more than half of patients required no further intervention after the initial procedure, while roughly one-quarter needed a single replacement. These findings suggest that septal buttons can provide meaningful symptom relief and medium-term control for many patients, although the possibility of revision or removal should be anticipated and discussed during preoperative counselling. 

Our findings align with previously published reports. Zaoui et al. [7] studied 45 patients treated with impression-moulded silicone buttons and noted significant improvement in crusting, epistaxis, obstruction, and whistling, with 91% patient satisfaction and 69% button retention at follow-up. Sapmaz et al. [5] compared surgical repair with septal button placement in 34 patients, showing that while surgical closure achieved higher quality-of-life scores, septal buttons offered partial symptom relief in those unsuitable for surgery. Earlier work by Facer and Kern [6] also described the long-term use of silastic buttons in more than 100 patients, reporting similar symptomatic improvement. Collectively, these studies and our audit suggest that silastic septal buttons remain a practical treatment option for selected patients, especially when surgical closure is not possible.

An important finding in our cohort was that over 80% of patients reported improved quality of life, and more than 90% said they would recommend the procedure to others. These patient-reported outcomes highlight the acceptability of silastic septal buttons and emphasize the value of incorporating patient satisfaction into the evaluation of prosthetic management strategies.

Documented complications in our series included infection in 7.8% of patients, granulation tissue in 5.2%, and migration in 2.6%. These figures align with earlier reports: Facer and Kern [6] noted local irritation and granulation in about 10% of cases and migration in up to 15%, while Zaoui et al. [7] observed removal in roughly one-fifth, most often due to discomfort, crusting, or infection. Overall, tolerance appears acceptable for most patients, though complications remain an important cause of removal or replacement. This underscores the need for careful counselling about the likelihood of ongoing management.

Several technical refinements have been described that may further influence outcomes. For example, Cohn et al. [8] reported the “sutured rosette” technique, developed to improve button stability and patient comfort, including in paediatric cases. Although this differs from our standard practice, it illustrates how insertion technique may affect tolerance and long-term retention.

This study has several limitations. It was retrospective, single-centre, and dependent on available clinical records. Missing information, such as incomplete documentation of perforation size and location, may have introduced bias. Outcomes were descriptive and based on routine follow-up without validated symptom scores, and retention rates may have been underestimated due to loss to follow-up. In addition, patient-reported outcomes were not collected with validated instruments and were partly obtained retrospectively, which may introduce recall or documentation bias. These factors limit the generalisability of our findings. Future studies should aim for larger, multicentre prospective cohorts with standardised outcome measures, including validated patient-reported tools, to clarify the long-term role of silastic buttons. Comparative studies of different button designs and insertion techniques could also help optimise tolerability and patient satisfaction.

Data availability

The data that support the findings of this study are available from the corresponding author upon reasonable request. Identifiable patient information cannot be shared publicly in accordance with institutional policy.

## Conclusions

Silastic septal button insertion appears to be a safe and practical management option for patients with symptomatic nasal septal perforations who are unsuitable for or decline surgical closure. While many achieved satisfactory symptom control without further intervention, a substantial proportion required replacement, adjustment, or removal. Given the retrospective, single-centre design and inherent limitations of this audit, the results should be interpreted with caution. Careful preoperative counselling remains essential to set expectations regarding the potential need for ongoing management. Overall, these findings support the role of silastic septal buttons as a viable treatment option in selected patients, pending confirmation from larger prospective studies with standardised outcome measures.
